# Factors influencing the resocialization of migrant older adults in China: a cross-sectional study

**DOI:** 10.3389/fpubh.2025.1511838

**Published:** 2025-04-15

**Authors:** Meijuan Cao, Yating Wang, Huiping Liu, Qiang Li

**Affiliations:** ^1^School of Nursing, Huzhou University, Huzhou, Zhejiang, China; ^2^School of Nursing, Hangzhou Normal University, Hangzhou, Zhejiang, China

**Keywords:** resocialization, migrant older adults, influencing factors, social adaptability, cross-sectional study

## Abstract

**Background:**

In the context of the further expansion of the aging population and the scale of migration, the issue of resocialization of migrant older adults has attracting attention due to its close association with their physical and mental health. This study aimed to identify the status and influencing factors of resocialization among migrant older adults under the guidance of the Social ecosystem theory framework.

**Methods:**

A cross-sectional study was conducted with a convenience sample of 977 migrant older adults from Hangzhou, China. The Chinese version of the Resocialization Scale was used to evaluate the resocialization degree of migrant older adults.

**Results:**

The total resocialization score of migrant older adults was 74.21 ± 9.08, with a range of 67.71–86.32% for each dimension, indicating that there is still significant room for improvement in the resocialization level of migrant older adults. Multiple linear regression analysis showed that age, education, monthly income, household registration, support from adult children, personality characteristics, resocialization willingness, satisfaction with the medical insurance system, satisfaction with the regional policies regarding older adults, and satisfaction with community construction were significantly associated with the level of resocialization.

**Conclusion:**

Community workers should develop individualized intervention plans according to the different circumstances of migrant older adults, integrate the strengths of Government, society, and the family, improve their ability to reintegrate into society, guide and support them, and promote their reasonable resocialization.

## Introduction

As rapid population aging accelerates and migration increases, more older adults have been migrating to other cities in pursuit of employment, retirement, or residing with children to care for grandchildren conveniently. Which has significantly increased the population of migrant older adults in recent years (1). This population of migrant older adults in China has now reached 18 million, accounting for 7.2% of the total migrant population, and is expected to maintain an average annual growth trend of 6.6% (2), according to the 2016 China Internal Migrants Dynamic Monitoring Survey. Owing to the dual fragile characteristics of “aging” and “immigratory” (3), migrant older adults are confronting certain challenges related to resocialization dilemmas after moving to new cities—including having new social networks, as well as linguistic communication, and living environment adaptations problems in their influx area. In addition, the restrictions imposed by the household registration system further impede the social integration of migrant older adults (4). As a result, the achievement of both social and spiritual values can be compromised, making this population prone to a sense of loneliness and loss (5). Therefore, helping migrant older adults integrate into their new environments as soon as possible and promoting their resocialization have become urgent issues.

The resocialization of migrant older adults refers to the process of these individuals actively changing their original values and lifestyles, as well as learning and establishing new values, and forming new sociocultural, societal, and interpersonal relationships to integrate into their new unfamiliar environments (6). This may entail undertaking activities such as active social participation, interpersonal interactions, re-employment, re-education, and other forms of social activity (7–11). Resocialization has been demonstrated to yield significant benefits for older adults, including improved physical and mental health, familial harmony and stability, and an enhanced sense of well-being (12–15). It can also enhance social connection in older adults, represents an important way for older adults to use, transform, and impart their knowledge and experience to achieve greater self-worth and contribute to society, and may be an important driver of age-appropriate development in aging societies (16, 17). Thus, resocialization not only represents one of the most important manifestations and criteria for measuring quality of life among older migrant adults, but also an important strategy for improving the well-being of this population in the context of active aging (18).

Surveys have shown that the majority of migrant older adults in China experience poor quality of life, with numerous obstacles and barriers to resocialization present during their reintegration into their new environments (19). Although previous studies have examined workplace-related resocialization and the resocialization of particular populations such as criminals, migrant older adults remain a socially marginalized and vulnerable population that has not received sufficient attention (20, 21). Most studies on the matter have assessed only one or a few aspects related to resocialization in older adults. For instance, Tomioka et al. assessed social participation among older adults by investigating the type and frequency with which this population participated in social activities, and found significant sex-based differences (22). A cross-sectional study of 4,823 older adults in China found that educational level and self-rated health also had significant impacts on the level of community participation among older adults (23). Kim et al. found that participation in social activities and good interpersonal relationships had a positive impact on resocialization in older adults who were migrating (8). However, no studies in the literature thus far have provided a holistic assessment of the resocialization of migrant older adults in China, and the factors influencing it.

Social ecosystem theory (SET) considers individual development to be nested within a series of interacting environmental systems and classifies them as micro-, meso-, and macro-systems, emphasizing interactions between individuals and their social environments and the impact on human behavior (24). It has been increasingly used to study personal and environmental factors related to psychology or behavior. For example, a previous study explored the determinants behind physicians’ intentions to accept online medical services and associated practices in micro-, meso-, and macro-systems, based on the SET theoretical framework (25). Inspired by SET, another study explored and confirmed that bidirectional social support for older adults is influenced by a combination of multilayered socio-environmental factors (26). Currently, most studies on the matter have explored the factors influencing resocialization from single viewpoints, and there is a persistent lack of systematic explorations of the factors influencing resocialization in migrant older adults based on multiple levels of SET. Based on the SET and existing research, this study intended to explore the major factors related to the resocialization of migrant older adults from the viewpoints of multi-layered socio-ecological micro- (i.e., individual factors and characteristics), meso- (i.e., familial factors), and macro-systems (i.e., societal and policy-related factors). This will hopefully provide a basis and reference for improving resocialization and general quality of life among this vulnerable population.

To achieve this, we applied the Resocialization Assessment Scale, which was developed specifically for migrant older adults (6), with the aim of assessing the current resocialization status of migrant older adults holistically, as well as systematically exploring the factors that influence it, based on SET. We hypothesized that various factors at the micro, meso, and macro levels affect the resocialization of older migrant adults.

## Materials and methods

### Design and participants

Referring to the data from the Seventh Population Census, Hangzhou, as the capital city and the largest city of population inflow of Zhejiang (the second largest province in the country in terms of the number of migrants), Hangzhou has attracted many inflows due to its well-developed economy. These migrants come from a wide range of sources of off-site domicile, covering both intra- provincial and extra-provincial inflows. This study used a cross-sectional design. Considering the purpose of the study as well as the readily accessible and feasibility for collecting samples, convenience sampling method was used in this study. From January to April 2020, a convenience sample of 977 migrant older adults were recruited from community health service centers, management centers, parks, public squares, and other major urban areas and economic development zones within Hangzhou, China.

In China’s retirement policy, the retirement age for men is 60 years, while that for women is 55 years (27). The household registration system represents a unique population registration management system in China. When a citizen is born, he or she is given a household registration status according to the administrative jurisdiction (city, county, or township) in which they were born. If an individual moves to another jurisdiction, he or she can choose whether to change his or her household registration. In this study, the definition of migrant older adults was: having lived in the place of data collection for ≥6 months, having non-local household registration, and meeting the age requirements outlined in the Chinese government’s retirement policy (2). The target inclusion criteria were as follows: (1) women aged ≥55 years or men aged ≥60 years; (2) those who are currently residing in the area, and duration of residence >6 months without local household registration; and (3) those who agreed to participate in this study and sign an informed consent form. The exclusion criteria were: (1) older adults with cognitive impairment, unconsciousness, or an inability to express their feelings clearly; (2) those who could not take care of themselves; and (3) those with severely impaired speech communication.

G*Power3.1.4 (Heinrich-Heine-Universität Düsseldorf, Germany) was used to determine that a sample size of 400 would be the minimum sample for this study, based on a one-way analysis of variance (ANOVA) with an effect size of 0.25, a statistical power (1–β) of 0.95, a significance level of 0.05, and five groups. A total of 992 older migrant adults were initially recruited, and the final sample size was 977—yielding a questionnaire completion rate of 98.5%.

### Measures

#### Socio-demographic questionnaire

Considering the influences on the resocialization of migrant older adults based on SET, our sociodemographic questionnaire was structured into three sections. At the micro level, it encompassed the basic characteristics of the participants (gender, age, educational level, household registration, monthly income, personality characteristics, reasons for immigration, self-rated health status, and resocialization willingness). At the meso level, it incorporated familial factors (marital status and support from adult children). At the macro level, it included community-related factors, (satisfaction with the community’s construction), and policy factors (satisfaction with the regional policies regarding older adults, household registration management, and medical insurance system).

Among these, “satisfaction with the household registration management” refers to the satisfaction of migrant older adults with the management of household registration in the place of inflow. In China, household registration management is still linked to residence, welfare, and primary health care. The “medical insurance system” refers to the satisfaction of migrant older persons with health insurance in their place of inflow. It includes satisfaction with the rate of reimbursement in the place of inflow, the types of illnesses covered by the reimbursement, and the adequacy of the extensive direct settlement function in other places. The “satisfaction with regional policies regarding older adults” refers to satisfaction with general older adults related policies in the inflow area that improve the quality of life, convenience, and satisfaction of the older adults, including satisfaction with the implementation of policies such as free public transport for the older adults in the place of inflow and aging-adapted renovation of public places.

#### Chinese older adult migrants resocialization scale

The Scale of Migrant Older Adults resocialization assessment was developed by Cao (5), and has been used to evaluate the degree of resocialization in older migrant adults. It includes four dimensions and 62 items: 20 concerning behavioral styles, 11 discussing societal roles, 14 assessing cultural integration, and 17 focused on interpersonal relationships. Each item had available responses of yes or no, and was scored as either one or two points. The final scores (i.e., the sums of the average scores for each dimension item and the product of the weighted coefficients of the corresponding dimensions) were converted into a percentage system on a scale ranging between 50 and 100 points. The Cronbach’s α coefficient for the present study was 0.892.

### Data collection

The investigators included two graduate nursing students and three community nurses. Prior to the official survey, all the investigators were required to undergo 1 week of training regarding the survey’s contents, the precautions to be taken, and communication skills tailored specifically to older adults. Data were collected by visiting community health service centers, community management centers, community parks, squares, and other places in the main urban areas and economic development zones of Hangzhou. Before data collection, the purpose, content, and requirements of the survey were explained to the study participants. After informed consent was obtained from the cohort, the survey questions were dictated by the investigator. The participants responded orally, and the investigator recorded their responses on the paper questionnaire. If the respondents were unable to understand some of the entries, the investigator reinterpreted the questions and asked them again. Immediately after the survey was completed, the investigators checked the data again for completeness and validity. The data were locked in cabinets to ensure confidentiality, and only the members of the research team had access to the data.

### Statistical analysis

Valid questionnaire information was inputted and double-checked using EpiData 3.1 software (EpiData Association, Odense, Denmark), and SPSS Statistics for Windows, version 26.0 (IBM Corp, Armonk, NY, USA) for all analyses. Before starting the formal analyses, we assessed the normal distribution of each variable. Tests of normality depending on the skewness and kurtosis coefficients indicated that if the absolute value of kurtosis was below 10 and the absolute value of skewness was below 3, the data, while not perfectly regular, could be considered reasonably close to normal. All the variables involved in this study have an approximately normal distribution, as evidenced by the absolute value of the maximum skewness of the variables involved in this study is 0.973, while the absolute value of the kurtosis is 1.903. These values indicate that the variables are normally distributed. Continuous variables were expressed as means ± standard deviations, and categorical ones were expressed as frequencies and percentages. The respondents’ total scores were found to be distributed normally. One-way ANOVA or Student’s t-tests were used to compare differences in resocialization among the participants’ demographic characteristics. The significant factors identified in the one-way ANOVA were selected as independent variables for multiple linear regression analyses to explore the factors affecting the resocialization of migrant older adults in China. The maximum variance inflation factor (VIF) involved in this study is 2.360, all of which are <10 and can be largely free of covariance. All the tests were two-sided, with a significance level of *α* = 0.05. Statistical significance was set at *p* < 0.05.

## Results

### Basic characteristics of the study participants

Out of the 977 participants, most of the migrant older adults surveyed were aged between 65 and 74 years (56.29%). There was a total of 615 women (62.95%) and 362 men (37.05%). Three-quarters (76.56%) of the migrant older adults had junior middle school or lower educational levels, most (89.25%) were married, and most (71.85%) were rural residents. The monthly income of most of the respondents (46.67%) was <200$ or between 200-500$ (38.38%). More than four-fifths (85.16%) had received support from their adult children, and about half (51.69%) considered their health status to be “moderate.” Less than half (45.65%) had clear intentions to resocialize, and the most common reason given as to why they immigrated was to take care of their grandchildren (64.59%). Concerning satisfaction with related policies, 53.12% were satisfied with the regional policies concerning older residents. In total, 50.15 and 38.59% of the respondents reported being satisfied with the regional medical insurance and household registration management systems, respectively (Table 1).

**Table 1:** One-way ANOVA or Student’s t-test of the different characteristics concerning resocialization among our cohort of migrant older adults in Hangzhou, China (*n* = 977).

Variables		N (%)	Resocialization total score (Mean ± SD)	t/F	*p*
Gender	Male	362 (37.05)	74.81 ± 9.79	1.544	0.123
	Female	615 (62.95)	73.86 ± 8.63		
Age (year)	55–64	404 (41.35)	77.31 ± 9.60	45.143	<0.001
	65–74	550 (56.29)	72.11 ± 8.12		
	≥75	23 (2.36)	69.96 ± 4.98		
Educational level	Primary & below	275 (28.15)	67.70 ± 6.43	374.89	<0.001
	Junior middle	473 (48.41)	73.11 ± 6.85		
	High & above	229 (23.44)	84.30 ± 7.12		
Marital status	Married	872 (89.25)	74.21 ± 9.08	2.931	0.054
	Unmarried/Divorced	53 (5.43)	72.62 ± 8.05		
	Widowed	52 (5.32)	71.82 ± 9.40		
Household registration	Rural	702 (71.85)	71.73 ± 7.83	−14.345	<0.001
	Cities and towns	275 (28.15)	80.57 ± 8.98		
Monthly income	<200$	456 (46.67)	68.73 ± 6.62	362.681	<0.001
	200–500$	375 (38.38)	76.46 ± 7.26		
	>500$	146 (14.95)	85.56 ± 6.71		
Support from adult children	Hardly/rarely	145 (14.84)	67.76 ± 6.18	241.685	<0.001
	Moderately	458 (46.88)	70.70 ± 6.31		
	Often/Always	374 (38.28)	81.01 ± 8.65		
Personality characteristics	Introversion	377 (38.59)	68.19 ± 5.99	367.007	<0.001
	Mixed personality	356 (36.44)	74.24 ± 7.30		
	Extrovert	244 (24.97)	83.48 ± 7.46		
Self-rated health	Good	405 (41.45)	78.41 ± 9.53	192.481	<0.001
	Moderately	505 (51.69)	71.60 ± 7.47		
	Bad	67 (6.86)	68.51 ± 6.75		
Reasons for immigration	Treat illness	46 (4.71)	71.58 ± 6.90	3.288	0.026
	Live out in retirement	280 (28.66)	73.52 ± 9.58		
	Work/farming	20 (2.05)	74.30 ± 7.62		
	Take care grandchildren	631 (64.59)	74.71 ± 9.00		
Resocialization willingness	Do not want to	442 (45.24)	68.00 ± 5.20	356.456	<0.001
	Do not know	89 (9.11)	74.51 ± 6.86		
	Willing to	446 (45.65)	80.31 ± 8.32		
Community’s construction satisfaction	Dissatisfied	58 (5.94)	67.51 ± 6.17	265.693	<0.001
	Moderately	646 (66.12)	71.15 ± 7.20		
	Satisfied	273 (27.94)	82.88 ± 7.63		
Regional Policy satisfaction for the older adults	Dissatisfied	19 (1.95)	65.50 ± 4.81	205.743	<0.001
	Moderately	439 (44.93)	69.26 ± 6.45		
	Satisfied	519 (53.12)	78.72 ± 8.69		
Household registration management system satisfaction	Dissatisfied	21 (2.15)	68.23 ± 6.17	134.696	<0.001
	Moderately	579 (59.26)	70.80 ± 6.99		
	Satisfied	377 (38.59)	79.79 ± 9.08		
Medical insurance system satisfaction	Dissatisfied	17 (1.74)	62.15 ± 3.06	271.544	<0.001
	Moderately	470 (48.11)	69.65±6.31		
	Satisfied	490 (50.15)	79.01±8.83		

### Resocialization status and univariate analysis

The mean (standard deviation) resocialization total score of the respondents was (74.21 ± 9.08). The maximum score was 95.07, and the minimum score was 54.23. The scores for each dimension ranged between 67.71–86.32%. Their specific values were 7.13 ± 1.21 for social roles, 28.51 ± 3.76 for interpersonal relationships, 10.87 ± 1.77 for behavioral styles, and 27.7 ± 5.45 for cultural integration (Table 2).

**Table 2:** Total resocialization score and scores for each dimension for migrant older adults Hangzhou, China (*n* = 977).

Dimension	Minimum score	Maximum score	Mean ± SD	Score rate
Behavior styles	7.92	15.85	10.87 ± 1.77	68.58%
Social roles	4.13	8.26	7.13 ± 1.21	86.32%
Cultural integration	20.46	40.91	27.70 ± 5.45	67.71%
Interpersonal relationships	17.49	34.98	28.51 ± 3.76	81.50%
Resocialization	54.23	95.07	74.21 ± 9.08	74.21%

One-way ANOVA and two independent-samples Student’s t-tests revealed significant differences in the total resocialization scores of the respondents related to factors such as age, educational level, household registration, monthly income level, support from adult children, self-rated health status, resocialization willingness, and reasons for immigrating; as well as levels of satisfaction with the regional community’s construction, the regional policies regarding older adults, household registration system, and medical insurance system (all *p* values <0.05). Among these, the respondents who reported being satisfied with the medical insurance system scored the highest, at 85.70 ± 9.57 (Table 1).

### Multiple regression analysis of the factors influencing resocialization

A multivariate linear regression analysis was conducted with the total resocialization score of the respondents as the dependent variable and the variables that showed significance in the univariate analysis as the independent ones. The results indicated that at the micro level, age, education level, monthly income, household registration, personality characteristics, and resocialization willingness were significantly associated with the level of resocialization. One unit increase in age was significantly associated with a decrease in resocialization (*β* = −0.058, *p* = 0.003). One unit increase in education level was significantly associated with an increase in the resocialization score (*β* = 0.274, *p* < 0.001). One unit increase in monthly income was significantly associated with an increase in resocialization (*β* = 0.155, *p* < 0.001). The resocialization for migrant older adults with an urban domicile were found to be higher than those for rural domicile (*β* = 0.081, *p* < 0.001). One unit increase in personality characteristics was significantly associated with an increase in the resocialization (*β* = 0.160, *p* < 0.001). One unit increase in willingness to resocialize was significantly associated with an increase in the resocialization (*β* = 0.209, *p* < 0.001).

At the meso level, support from adult children and satisfaction with community’s construction was significantly associated with the level of resocialization. One-unit increase in support from adult children was significantly associated with an increase in the resocialization (*β* = 0.066, *p* = 0.008). An increase of one unit in satisfaction with community’s construction was significantly associated with an increase in resocialization score (*β* = 0.085, *p* = 0.001).

At the macro level, satisfaction with regional policies regarding older adults and medical insurance system is significantly associated with the level of resocialization. One-unit increase in satisfaction with regional policies regarding older adults was significantly associated with an increase in resocialization (*β* = 0.056, *p* = 0.021). Similarly, one unit increase in satisfaction with the medical insurance system was associated with a significant increase in resocialization score (*β* = 0.127, *p* < 0.001) (Table 3).

**Table 3:** Linear regression analysis of the factors influencing resocialization among a cohort of migrant older adults in Hangzhou, China (*n* = 977).

Variables	*B*	SE	*β*	*t*	*P*	95.0% CI	
(constant)	41.032	2.466		16.642	0.000	36.194	45.870
Age (ref:55–64)	−0.993	0.332	−0.058	−2.992	0.003	−1.645	−0.342
Education level (ref: primary & below)	3.053	0.280	0.274	10.915	0.000	2.504	3.602
Household registration (ref: rural)	1.635	0.477	0.081	3.431	0.001	0.700	2.570
Monthly income (ref: <200 $)	1.958	0.347	0.155	5.634	0.000	1.276	2.640
Support from adult children (ref: hardly/rarely)	0.872	0.326	0.066	2.679	0.008	0.233	1.511
Personality characteristics (ref: introversion)	1.847	0.306	0.160	6.035	0.000	1.246	2.447
Self-rated health (ref: good)	0.602	0.359	0.040	1.674	0.094	−0.103	1.307
Resocialization willingness (ref: do not want to)	1.993	0.230	0.209	8.664	0.000	1.541	2.444
Reasons for immigration (ref: Treat illness)	−0.006	0.182	−0.001	−0.032	0.975	−0.362	0.351
Community’s construction satisfaction (ref: dissatisfied)	1.433	0.420	0.085	3.408	0.001	0.608	2.258
Regional Policy satisfaction for the older adults (ref: dissatisfied)	0.945	0.408	0.056	2.318	0.021	0.145	1.745
Household registration management system Satisfaction (ref: dissatisfied)	0.536	0.404	0.031	1.327	0.185	−0.257	1.328
Medical insurance system satisfaction (ref: dissatisfied)	2.167	0.411	0.127	5.277	0.000	1.361	2.973

*R = 0.832, R2 = 0.687, F = 166.095, P < 0.001.*

## Discussion

This study showed that the total resocialization score of the migrant older adults in Hangzhou, China was 74.21 ± 9.08, with a range of 67.71 -86.32% for each dimension, indicating that there is still much room for improvement in the resocialization level of migrant older adults. Only 45.65% of the respondents indicated a clear willingness to resocialize, meaning that considerable scope remains for improvement overall. This may be because of general neglect of migrant older adults by society that has resulted in a lack of social recognition for this group (28, 29), along with a decreased sense of self-identity, low levels of inclination to resocialize, and weak overall willingness to resocialize (30). Additionally, compared to local older adults, migrant older adults generally have more limited access to social resources, which may restrict their ability to resocialize to certain extents (31).

Among the four dimensions in our survey, our cohort scored highest in the societal role (86.32%) and interpersonal relationship dimensions (81.50%) of resocialization, indicating that migrant older adults are able to adjust to shifts in their societal roles to a certain extent, and initially establish social networks in their new regions. This may be because of the ethos of traditional Chinese family culture, wherein middle-aged and older family members often play the roles of familial caregivers (32). Most of the migrant older adults in this study were female, had lower educational levels, had rural household registrations, and migrated mainly to take care of their grandchildren. Their daily lives therefore tended to center on household services, where they had clear expectations of their own roles. This likely enabled them to adapt to their changing roles and better realize their values within their families. Similar to the study by Sun et al., in which the migrant older adults were able to participate in basic social activities in the inflow area, with friends and family members being the main targets of social interactions (33). Owing to similar experiences and characteristics, groups of older adults who migrate to care for their grandchildren often converge in terms of their life patterns, confront similar social and general challenges, and are more inclined to cultivate in-group support and assist one another (15). In addition, research also indicates that migrants whose primary motivation for relocation is employment tend to migrate with their friends and family members from the same area of origin (34), which not only provides them with more social network connections but also strengthens their sense of social role identity, which in turn improves their adaptive capacity and enables them to better resocialize in the place of inflow (35).

However, among the four dimensions in our survey, our respondents scored poorly in the behavioral participation (68.58%) and cultural integration dimensions (67.71%) in their new region, with low levels of participation in volunteer activities and re-employment activities. Only 28.4% of our respondents reported that they participated in activities or gatherings in their neighborhoods, which is consistent with the findings of Xing (36) and Matsue (37). This indicates that migrant older adults often have low levels of participation in activities within their new regions, which may be due in part to local language and cultural barriers. Most of the participants in this survey indicated that they were unwilling to learn a different language or understand local customs in order to adapt to their new environment. It is possible that learning to speak the new language of the region of relocation becomes more challenging for older adults because of the progressive loss of cognitive and active learning abilities, as well as general declines in intellectual capacity brought about by age and declining brain function (38). Due to large socio-ecological differences, such as the diversity of linguistic language varieties and cultural differences, whether working or caring for children, migrant older adults face resocialization barriers. Furthermore, the majority of the older adults surveyed in this study had migrated because of their familial roles related to caring for their grandchildren. As a result, their daily activities were primarily centered on their children and grandchildren, with the time, types, and locations of these activities remaining relatively fixed and limited. This likely restricted their abilities to participate in social activities to a certain degree (15).

As we hypothesized, this study demonstrated that age, educational level, household registration, monthly income, and willingness to resocialize represent micro-level systemic factors that affect resocialization among migrant older adults; support from adult children and satisfaction with community-building represent important meso-level factors; and that regional policies regarding older adults, and medical insurance systems represent important macro-level factors.

At the micro level, this study showed that age is negatively correlated with the level of resocialization. This is similar to the findings of Moore et al. (39), who found that, as age increases, daily activities tend to become more restricted in older adults, and their willingness to resocialize decreases. Tang’s study showed that the educational levels of older adults are positively related to their level of resocialization (40), which was similar to our findings. A higher educational level indicates that individuals have strong comprehension abilities, in turn conferring higher social adaptability that may allow them to overcome social and cultural changes (41). Higher levels of migrant resocialization are generally found in urban households compared to rural ones, which may be explained by the greater allocation of social resources and services in urban regions compared to rural ones (42). Consequently, migrant older adults living in urban households may have greater access to more social benefits and favorable conditions for resocialization.

A previous study revealed that lower-income older adults have a low willingness to resocialize and low levels of participation in social activities and potential barriers to cultural integration (43), which may be because domestic competition is intense, and high-paying and relatively less laborious jobs tend to be associated with higher educational attainment. In contrast to higher-income migrant older adults, lower-income migrant older adults tend to be less educated, which limits their opportunities to resocialize and the performance-to-price ratio of resocialization is therefore less high. Tomioka found that changes in daily social activities among older women were associated with participation in different types of social activities (22), but not among men. In that study, older women who participated in different types of social activities showed higher levels of daily activities and degree of socialization. However, this study found no statistically significant differences between our participants in terms of sex and resocialization; therefore, the correlation between the degree of resocialization and sex among migrant older adults merits further exploration. This study was conducted during the COVID-19 pandemic, so our cohort of older migrant adults may have also generally lacked opportunities for resocialization owing to pandemic-related restrictions.

At the meso level, we found that support from adult children represented an important familial factor that affected resocialization in our cohort. According to this research, adult children can offer more care and assistance to older adults later in life, fostering a positive family atmosphere and also providing important emotional support (44). Consequently, adult children should realize that their concern has an important impact on the integration of their parents into new environments, provide more care and support for their migrant parents in their daily lives, understand their parents’ needs, and provide them with appropriate help and guidance to promote the resocialization of migrant older adults.

The construction level of community and surrounding related infrastructure also represented an important community factor affecting the resocialization of migrant older adults, which is consistent with the results of Liu et al. (45). This is likely because the community represents the primary places where migrant older adults live, and each has its own particular regional, functional, and emotional characteristics. More compatible and aging-friendly community constructions tend to encourage more participation from migrant older adults, which helps to promote their resocialization. Therefore, particular attention should be paid to older migrants in rural regions with lower levels of education and income, and community construction that is friendlier to migrant older adults should be on the agenda. Community healthcare workers and administrators should pay attention to the behavior and cultural integration of older migrant adults in these areas—particularly in terms of participation in volunteer and re-employment activities—as well as the integration of language and customs, in order to encourage and provide opportunities for them to learn the local language and acquire new skills. Re-employment platforms should also be built to actively promote the development of re-employment projects and positions for migrant older adults.

At the macro level, this study found that satisfaction with the regional security and medical insurance policies for older adults promotes good social adaptability and resocialization among migrant older adults, which is similar to the findings of previous related studies (46, 47). Likely owing to the limitation of the existing household registration system, migrant older adults may not be able to enjoy the same security and medical services as they did in their previous regions of residence. This can result in fewer connections for them within their new regions, as well as less sense of belonging. The government should not only pay attention to the impacts of regional economic development on migrant older adults, accelerate the development of medical insurance and public services, and provide multi-level and multi-functional social and public services for them—but should also reform the household registration system so that these individuals can enjoy the same welfare benefits as they did in their previous regions.

### Limitations

This study was subject to several key limitations worth noting. First, although these findings were obtained from a large sample, they are only representative of Hangzhou. Therefore, future studies are warranted to expand the population sampled and the range of sampling, as well as conduct a comparative analysis of different regions to obtain more representative and robust results. Second, although our research is grounded in the theory of SET and considers the impact of numerous systematic factors on the resocialization of older migrant adults, certain potential factors may also impact this process, owing to the intricacy of resocialization. In the future, qualitative research can be combined to better understand the demands associated with the resocialization behaviors of migrant older adults and develop successful intervention programs. Furthermore, although we have taken some measures to avoid endogenous bias, the causal relationship between the relevant factors and resocialization could not be determined on the cross-sectional survey, and resocialization is a dynamic process. Therefore, future studies need to adopt a longitudinal design to track and understand the changes in the resocialization behavior of migrant older adults and the relationship between the relevant predictors and the resocialization of migrant older adults.

## Conclusion

Overall, our results showed that there is significant room for improvement in the level of resocialization of migrant older adults. The factors affecting the level of resocialization among older migrant adults also differ. Age, educational level, household registration, monthly income, personality characteristics, resocialization willingness at the micro level, support from adult children, community’s construction status at the meso level, and regional policies regarding older adults and the medical insurance system at the macro level represented the main factors affecting resocialization in this population. Therefore, future research should focus on developing cost-effective interventions to assist older migrant adults with integrating into local demographic groups in their new regions as soon as possible. This will likely help them to increase their feelings of self-worth, improve their quality of life and social well-being, and further promote their resocialization.

## Data Availability

The raw data supporting the conclusions of this article will be made available by the authors, without undue reservation.
